# Biographical Notice of the Late Josiah Foster Flagg, M. D., D. D. S.

**Published:** 1854-10

**Authors:** 


### BIOGRAPHICAL NOTICE OF THE LATE JOSIAH FOSTER FLAGG, M. D., DENTIST, OF BOSTON.

Dr. Josiah Foster Flagg, was born in Boston, January 11th, 1789. His father, Dr. Josiah Flagg, was long known as the "Boston Dentist," as he was almost the only person who confined his whole attention to the profession—dentistry being at that time in its infancy.

Dr. F. was the eldest of the family. He received but an indifferent early education, but improved his few advantages so well, as to be prepared to enter as a student of medicine under the tutelage of Dr. J. C. Warren, in 1811. The circumstances under which he commenced his studies were very discouraging, as he had but few friends, no pecuniary resources, and from various causes, his prospects were indeed gloomy. He sustained himself under these trials with unflinching courage, and sought, by unwearied industry, to discharge with fidelity the heavy duties resting upon him.

Dr. Warren, in allusion to Dr. F., at this period, states that "he was well educated as a surgeon having devoted a year more than usual to his preparatory studies." "He discovered at an early period, great mechanical ingenuity and mental activity."

In 1813, he undertook, in connection with Dr. Warren, the publication of a work on "The Arteries;" the first of the kind ever published; as the custom had hitherto been to describe the larger arteries with but little more minuteness than the smaller. The engravings were the work of Dr. F.'s own hand, and were executed with such remarkable skill, as to elicit the highest encomiums from the best judges. The work had a great sale, and in a short time the edition was exhausted; a second was contemplated, but from some cause not issued. The book is now rare; but, for beauty and accuracy of design and execution, will compare most favorably with the best works of the present day. A few years afterward he prepared for Dr. Warren, drawings for a publication called "Comparative Views of the Nervous System."* Dr. W. says, "the representations of the anatomy of the leech, lobster, oyster and centipede, were beautifully and accurately done, and would, I believe, do credit to any artist of the present day, for these were executed between thirty and forty years ago." "At an early period, he (Dr. F.) contrived various surgical instruments, particularly the bone-forceps, which almost produced a revolution in the operative surgery of the bones. This was

*See Reports Mass. Med. Soc. vol. 3, p. 307.

long before Liston's forceps, or any other that I know of."*

In 1821, Dr. F. published, in the N. E. Med. Journal, vol. 10, p. 38, a description of his improvements on Desault's apparatus for fracture of the thigh bone, with observations on the treatment, etc. This apparatus was introduced by Dr. W. into the Massachusetts General Hospital, and has been used in that and other institutions ever since, as the most perfect thing of the kind yet discovered.

After graduating in 1815, Dr. Flagg practiced for sometime in Uxbridge, Mass., but was persuaded by Drs. Warren and James Jackson to remove to Boston, where he commenced the practice of dentistry. About this time he married Miss Mary Wait, daughter of Mr. T. B. Wait, of the well known firm of Wait & Lilley, printers and publishers. This union proved a most happy one. Dr. F.'s business now increased so rapidly, that he was compelled to relinquish almost entirely the general practice of medicine, though his inclination still led him to continue the treatment of disease in its chronic forms. For a long period he was almost the only person in Boston who could, with propriety, be termed a "surgeon dentist,"† as his cotemporaries, Drs. Randall and Greenwood, confined their attention to mechanical dentistry, leaving to him the more difficult surgical department.

In the fall of 1833, Dr. F. commenced, in connection with Dr. N. C. Keep, the manufacture of mineral teeth. In a note on the subject, Dr. K. says, "Dr. Flagg and myself had felt the necessity of a more durable article than the hippotamus, cows or human teeth. Even French porcelain-teeth, of which there was a large assortment, though incorruptible, were unsatisfactory, because unnatural. After careful examination, we concluded that as yet nothing had been produced adequate to the wants of the profession or the community.

"At that time there were several dentists, who made and used teeth called by various names, such as 'mineral-paste-

* Letter from J. C. Warren, M. D., Feb. 24, 1854.

† I have since learned that Dr. T. W. Parson, M. D., was practicing in Boston at that time.

teeth,' 'composition-teeth,' 'metallic-teeth,' etc. Feeling confident that I understood the views of Dr. Flagg, and that he, as well as myself, would be willing to pay well for knowledge of any important improvement in our art, I made personal application to one of the above, offering to pay a reasonable portion of the expense, the art had thus far cost to those initiated into its mysteries.

"The answer received was short, 'I have got the art and it shall live and die with me!' No greater stimulus than this rebuff was required by Dr. F. or myself, to incite us to renewed exertions, which, we determined should not cease, but with success equal at least to that of our rival. A charlatan made his appearance soon after, who professed to understand the whole subject. He exhibited a few specimens, but would not impart the great secret and practical demonstration, unless the very moderate sum of $1000 was first secured.

"After devoting ourselves exclusively to this pretended instructor, day and night for about six weeks, my own house having been set on fire, and that of Dr. F. narrowly escaping a similar fate, we concluded that it would be best to pay off our *humbug*. Availing ourselves of such general principles, as we had obtained respecting the materials used by him, we began anew our career, for as yet we had not made a tooth which satisfied us. We received aid from our friends, the chemists, who prepared for us pure colors, and from mineralogists, who procured excellent felspar. We planned our course on the principles of science, and kept careful records of our progress. Our success was greater than we expected. In the course of six months we had the pleasure of knowing that we could make the best mineral teeth."

After this time Dr. F. continued his experiments in this department of his business, with untiring zeal, until a short period before his decease, never resting satisfied with his attainments, but ever striving to improve; his aim being constantly to elevate every department of his profession to the extent of his ability.*

In 1844–45 he conceived the idea of drilling into the nerve-chamber, in order to prevent the ill consequences arising from filling over the exposed or diseased nerve. After testing the operation for between two or three years, he published the result of his observations in the Boston Medical and Surgical Journal, Jan. 27, 1847, with drawings illustrating the mode of performance.

In 1846 Dr. F. became involved in the somewhat famous ether controversy, taking an early and decisive stand against the legality of patenting such a discovery, and that, as a patent medicine, it should be used by professors of the medical school in the Massachusetts General Hospital, in violation of a by-law of the Massachusetts Medical Society. Though severely censured in some quarters, for the course he took, the justness of his views was at length acknowledged, and subsequently, Dr. Jackson freely gave the whole thing to the public.*

In 1839 Dr. F. became interested in the almost unknown doctrine, at that time, of homeopathy, and the decided stand he took in favor of *the new system*, cost him the friendship of some of his oldest and best friends. He was the first to introduce it to the notice of the Boston public, and to the last of his life was a firm believer in the truths of its tenets. In stating the reasons for this change of opinion, he remarked that he was at first decidedly opposed to it, from the apparent absurdity of its teachings; and it was not, until he had thoroughly tested it by experiment, and witnessed the beneficial effect of the treatment in numerous obstinate cases, acute and chronic, that he gave his adherence to it. Might not his mode of investigating the subject serve as a lesson to many in the profession, who, after trying a few of the remedies, without properly understanding their use, or the mode of selection, proceed to denounce the whole system in the most dogmatic manner? After spending some months in the study of homeopathy, he carefully collected the symptoms of some cases and submitted them to the inspection of experienced homeopathic practitioners in New York and Philadelphia, who were his personal friends, and adminstered the remedies according to their directions. This course he pursued for some time, not trusting his own judgment in the selection of the medicines. After watching their effects in a large number of well marked cases, he became convinced that there was something more than "imagination" in the beneficial results that followed their use. In the space of a few years he collected the records of near three hundred cases, mostly of chronic diseases, which were treated by himself. The results of several were published in the periodicals of the day. He confined his attention almost exclusively to the treatment of chronic complaints, as he had not sufficient leisure for those of an acute nature. The success that attended his treatment brought a large number of those suffering from long protracted disease, to his door; but finding that his own health failed from the pressure of business and close confinement, he was obliged to relinquish, in a great measure, the numerous applications made to him.

The School of Design for Women, in Boston, was among the latest of his public efforts. It is founded on the plan of a similar one in Philadelphia. Having visited that school, and becoming interested in its object, he conceived the idea of establishing one in his native city, and had the satisfaction of living to see it placed on a firm basis, as the state has recognized its utility and testified its approbation, by an annual grant of $1,500 for three years.

As one of the pioneers of dentistry, in this country, Dr. F. deserves especial consideration. He ever regarded dentistry as one of the noblest of the professions; and it is no wonder that he watched carefully, and censured freely, anything calculated to lower it in the eyes of the public.

He was eminently a *benevolent man;* not of that class who do good for the praise of men. He ever labored in a quiet, private way, to benefit those who required and deserved assistance; and many, now prosperous in life, can look back with the most grateful emotions to the time, when, poor and friendless, they found in "the good doctor" a friend ever ready to assist with counsel and purse, their early struggles with the world.

Having tasted the bitter cup of poverty and disappointment, and knowing by sad experience the trials of striving against hope, he could the more readily sympathize with those, who, placed in similar circumstances, needed some one to encourage and advise them. Although his kindness sometimes met with ungrateful returns, he continued unwearied in good works, and never permitted anything to shake his confidence in, nor weaken his benevolent regard for his fellow man.

Of remarkably bland, gentlemanly address, and easy of access, he won the confidence and esteem of all who knew him. His probity was proverbial, his moral character of the highest tone, and his views liberal and enlarged. Accustomed to the free expression of his opinions, he rebuked presumption and imposture wherever he found it; and as he would never praise unless the object were really worthy, neither would he suffer any personal consideration to affect his estimate of moral or professional worth.

His last illness was but the crisis of a chronic disease. For years he had suffered from that terror of professional men—dyspepsia; and within the last few years of his life, each season found him more feeble than the preceding. Originally of a delicate constitution, the close confinement and laborious duties of his profession, increased the tendencies to gastric difficulties, year by year.

After suffering most intensely from a neuralgic affection of the stomach, for some months, and which finally increased to such a degree that not even the lightest nourishment could be borne, accompanied by extreme emaciation of body and depression of spirits, his strength yielded, and he "became immortal," departing this life December 20th, 1853.—*American Journal of Dental Science.*

